# Is surgical excision necessary for the treatment of Granulomatous lobular mastitis?

**DOI:** 10.1186/s12905-017-0412-0

**Published:** 2017-07-24

**Authors:** Young Duck Shin, Sung Su Park, Young Jin Song, Seung-Myoung Son, Young Jin Choi

**Affiliations:** 1 Department of Anesthesiology, Chungbuk National University Hospital, Chungbuk National University School of Medicine, 410 Sungbong-ro, Heungdeok-gu, Cheongju, 28644 Chungcheongbuk-do South Korea; 2 Department of Surgery, Chungbuk National University Hospital, Chungbuk National University School of Medicine, 410 Sungbong-ro, Heungdeok-gu, Cheongju, 28644 Chungcheongbuk-do South Korea; 3 Department of Pathology, Chungbuk National University Hospital, Chungbuk National University School of Medicine, 410 Sungbong-ro, Heungdeok-gu, Cheongju, Chungcheongbuk-do 28644 South Korea

**Keywords:** Female, Granulomatous mastitis, Autoimmune disease, Breast diseases, Lobular mastitis

## Abstract

**Background:**

We aimed to investigate the role of surgical excision in treating granulomatous lobular mastitis.

**Methods:**

We performed a retrospective chart review of patients with granulomatous lobular mastitis treated from March 2008 to March 2014. We analyzed clinical features and therapeutic modalities and compared the patient outcomes based on treatment.

**Results:**

During the study period, a total of 34 patients were diagnosed with granulomatous lobular mastitis and treated. Initial treatments included wide excision (18), oral steroids after incision and drainage (14), and antibiotic therapy (2). The patients receiving only antibiotic therapy showed no improvement after 1 month and wide excision was then performed. Wide excision resulted in nine case of delayed wound healing with fistula. These patients were treated with oral steroids for 1.5-5 months, with subsequent improvement. Overall, 11 out of 20 patients who had underwent wide excision showed improvement without additional treatment. Fourteen patients who had initially received oral steroids for 1 to 6 months (average, 2.8 months) after incision and drainage showed complete remission. During the median follow-up period with 45.5 months (range, 22–98 months), six patients (17.6%) experienced recurrence. Wide excision group experienced recurrence in five (25%) and steroid and drainage group experienced recurrence in one (7.1%). All six recurrences responded to additional steroid therapy for average 3.5 months. Most wide excision group left extensive breast scarring with deformation that was not in steroid and drainage group.

**Conclusions:**

Wide excision resulted high recurrence than steroid and drainage group and left extensive scarring. Steroid therapy with or without abscess drainage may be the first choice of treatment for majority cases with granulomatous lobular mastitis.

## Background

Granulomatous mastitis (GM) is a term for granulomatous inflammation of the breast that is idiopathic or occurs in association with diseases such as tuberculosis (TB), sarcoidosis, and parasitic infection. There are reports of a rare form of unknown cause that results in noncaseating granulomas and microabscesses localized to the breast lobule. This type is referred to as idiopathic granulomatous lobular mastitis or granulomatous lobular mastitis (GLM).

GLM was first described by Kessler et al. in 1972 [1]. The incidence of GLM is unclear, and most reports regarding the condition are case series. It is a rare disease that occurs mostly in women of childbearing age [2, 3]. Its reported causes include hypersensitivity to lactation products extravasated into the breast tissue, localized breast trauma, subclinical infection, and autoimmune processes. It may also be associated with mammary duct ectasia and corynebacterial infection. GM from TB forms caseating granulomas that are accompanied by a great degree of fibrosis and necrosis, and thus it can be distinguished from GLM based on histopathology [4].

Symptoms of GLM include fistula, abscess, and a palpable mass in the breast. In many cases, patients present with an ill-defined breast mass with radiographic features suggestive of malignancy. In such cases, it is absolutely necessary to perform a biopsy, to differentiate the mass from a malignant lesion. A biopsy of GLM often shows noncaseating granulomas centered on the breast lobules within the breast parenchyma. Neutrophilic microabscesses are frequently present.

Opinions regarding the cause of GLM vary, and there is controversy about the optimal treatment. Although many studies have addressed this issue and various treatment modalities have been reported, this is still an open question. For this reason, we analyzed the medical records of 34 patients with GLM in order to determine the role of surgical excision and steroid therapy in the treatment of GLM.

## Methods

Our study was approved by the Chungbuk National University Hospital Institutional Review Board. We performed a retrospective chart review of patients with a histological diagnosis of GLM treated at our hospital’s breast clinic from March 2008 to March 2014. Thirty four patients were identified among those being treated for benign breast disease. Baseline characteristics, clinical features, and treatments were analyzed. Baseline characteristics included age at diagnosis, history of pregnancy and lactation, history of TB or autoimmune disease, oral contraceptive use, smoking status, and family history of breast cancer. Clinical features included primary symptoms, the presence of a mass, abscess, or fistula, location of the lesion within the breast, and nipple involvement. The results of diagnostic studies including culture studies, mammograms, ultrasounds, and histologic examinations were evaluated. The initial treatment modality, duration of, and response to this treatment, disease recurrence, changes in treatment plan, total treatment period, and treatment complications were recorded for each patient. Disease recurrence was defined as the reappearance of fistula, abscess, or mass on the ipsilateral breast.

## Results

### Clinical characteristics

During the study period, 4016 cases of ultrasound - guided breast biopsy and 1770 cases of operation for benign breast disease were performed. Of those, a total of 34 cases (0.84% among biopsied patients and 1.92% among operated patients) were definitively diagnosed with GLM and treated accordingly. Table 1 shows the clinical characteristics, treatment and outcome of 34 patients with GLM according to initial treatment. The median age of patients was 37 (range, 24 - 57) years. Thirty two patients were of reproductive age. Among these patients, the mean duration of their last childbirth to diagnosis was 38 months (range, 1 - 72 months), and the mean duration from the end of lactation to diagnosis was 25 months (range, 1 - 60 months). Nine patients (26.5%) were smokers and 5 patients (14.7%) were taking oral contraceptives at the time of diagnosis. One patient had a family history of breast cancer. None of the patients had a history of TB or autoimmune diseases.

**Table 1:** Clinical characteristics and presentation, outcome of 34 patients with GLM according to the treatment modalities

	Wide excision (*N* = 20)	Steroid and drainage (*N* = 14)
Age (year, median)(range)	33 (24-46)	38 (28-57)
Presentation since last delivery, month (range)	16 (2-72)	10 (1-24)
Breast fed in the last 6 months (%)	3 (15%)	2 (14.2%)
Use of oral contraceptive (%)	3 (15%)	2 (14.3%)
Smokers (%)	6 (30%)	3 (21.4%)
Clinical presentation		
painful mass	12	7
pain and erythema	6	5
fistula with discharge	2	2
Mean mass size, cm (range)	5.8 (1.5-8)	4.2 (2.4-6)
Over two quadrants Involved or nipple involved (%)	8 (40%)	6 (42.8%)
Recovery time, month (range)	4.1 (1-7.5)	2.8 (1-6)
Additional steroids needed	9	0
Follow up time, month (range)	35 (16-98)	42 (22-67)
Recurrence (%) (month after initial treatment)	5 (25%) (6-12)	1 (7.1%) (7)
Treatment of recurrence (duration, months)(range)	Steroid therapy (1.5-4)	Steroid therapy (1.5)

Nineteen patients presented with a palpable mass, 11 with breast pain and erythema, and four with fistula and breast discharge. The mean lesion size was 3.5 cm (range, 1.5 – 8 cm) and 11 patients had infiltration into more than two breast quadrants. The disease had progressed to the subareolar area in 14 patients.

**Fig. 1:**
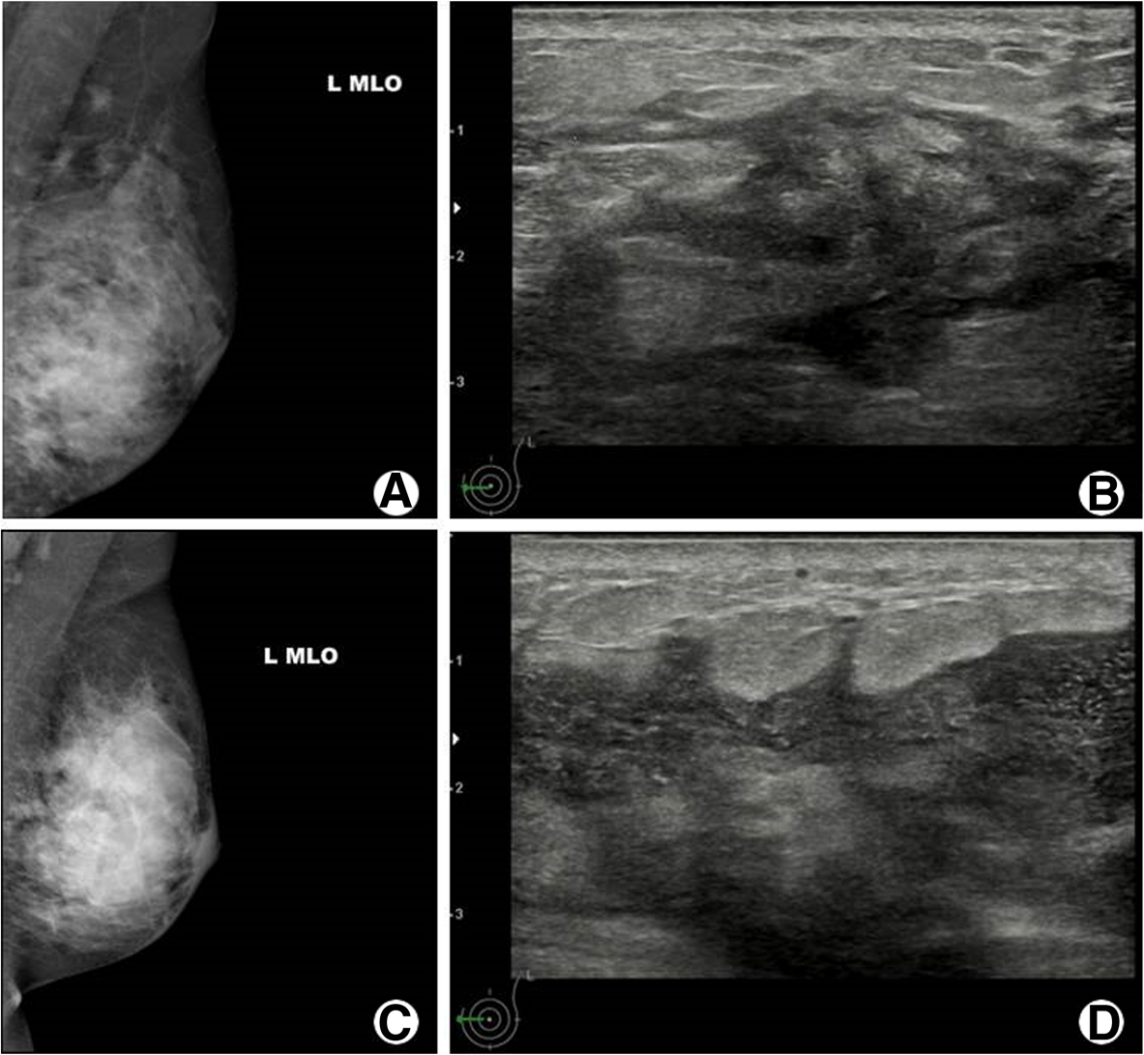
Findings of mammography and ultrasonography of two patients with granulomatous lobular mastitis. Patient 1. There was asymmetric increased density on *left lower* breast (**a**) and ultrasonography reveals a hypoechoic and irregular shaped mass with ill defined margins (**b**). Patient 2. Oblique view of *left* breast demonstrated diffusely increased asymmetric density on *left* breast and enlarged axillary lymph nodes (**c**). Ultrasonography showed irregular hypoechoic mass with internal debris with skin thickening, consistent with abscess (**d**)

Mammogram results showed an ill-defined asymmetric density in all patients. Breast ultrasound revealed microabscesses in the breast parenchyma appearing as irregular hypoechoic masses with internal debris in 26 patients, and a hypoechoic nodular structure with no distinct mass in 8 patients (Fig. 1). No patient underwent an MRI examination.

**Fig. 2:**
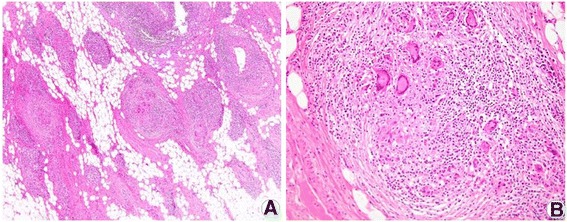
Pathologic findings of granulomatous lobular mastitis. Wide excision biopsy specimen demonstrating the non-caseating granulomas involving mammary lobules, with multinucleated giant cells (**a**. hematoxylin and eosin, X40) and infiltration of lobules with neutrophil and lymphocytes (**b**. hematoxylin and eosin, X200)

All patients underwent ultrasound-guided core needle biopsy with microscopic examination of the tissue specimen, and all diagnosed as GLM. Histopathologic findings included noncaseating granulomatous inflammation characterized by scattered granulomas composed of epithelioid histiocytes and giant cells accompanied by lymphocytes, neutrophils, plasma cells, and eosinophils (Fig. 2). Acid-fast bacilli (AFB) staining was performed on all specimens to rule out TB infection, and was negative in each case. A polymerase chain reaction test for Mycobacterium tuberculosis (TB-PCR) was performed in 32 patients and was negative in all cases. Gram stain and microbial culture using open pus from wound were performed on 21 cases with abscess and resulted as negative in all cases. Rather, methicillin-resistant *Staphylococcus aureus* was cultured on one case who experiencing relapse, after completion of steroid treatment. She was managed with antibiotics.

### Treatment and outcome

The initial treatment was antibiotic therapy for two patients, wide excision with clear margins under general anesthesia for 18 patients, and incision and drainage under local anesthesia followed by steroid therapy for 14 patients. Steroids were initially administered with 20 mg/d of prednisone (PD), and if the symptoms improved the dose was reduced to 10 mg/d.

Two patients who initially treated with antibiotics did not show improvement after 1 month, and they underwent wide excision. Among the 18 patients who initially underwent wide excision, nine experienced delayed wound healing with fistula formation involving the excision scar. As a result, these patients also received postoperative steroid therapy, with a mean duration of 3.5 months (range, 1.5 - 5 months). Among the 14 patients who received incision and drainage followed by steroid therapy, the mean duration of steroid treatment was 2.8 months (range, 1-6 months). The lesion resolved completely in 11 of the patients. One patient who received incision and drainage followed by 3 months of steroid treatment experienced a reduction in lesion size. She wanted complete removal of lesion and percutaneous vacuum-assisted excision of the affected tissue wad performed, subsequently showed a complete response. Two patients who received incision and drainage followed by 1 month of steroids showed symptom improvement and had no pain or mass after steroid treatment, but remained microabscesses on ultrasound. At the request of each patient, steroid use was terminated and care consisted of observation only. Among the 23 patients who received steroid therapy, two experienced weight gain. None of the patients developed other side-effects associated with steroid use.

The median follow-up period was 45.5 months (range 22 - 98 months), and during this time six patients showed recurrence. Among 20 wide excision group, five (20%) experienced recurrence of disease. They were cases who received additional steroid therapy for delayed wound healing after wide excision. Among drainage and steroid therapy group, one (7.1%) experienced recurrence.

The time to recurrence took place at 6 to 12 months, following completion of the initial treatment. Most recurrences developed as skin abscess and fistula at the excision or draining site and one patient developed a mass in a breast quadrant that was not part of the initial presenting site. All recurrences showed improvement after treating steroid with median duration of 2.5 months (1.5 - 4). The two patients followed only with observation despite a lack of complete treatment response were followed for 36 and 40 months. During this time, the patients did not develop new symptoms, and only heterogenous hypoechoic lesions without microabscess remained on their last ultrasound results.

**Fig. 3:**
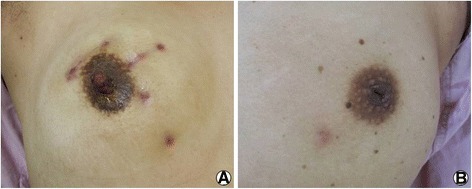
Outcome of granulomatous lobular mastitis according to treatment. Wide excision resulted hypertrophic scarring and breast deformation (**a**), while the patients who received incision and drainage followed by steroids had scarring limited to the drainage tube inserted site (**b**)

Eighteen of the 20 patients who underwent wide excision showed extensive hypertrophic scarring and breast deformation, while the patients who received incision and drainage followed by steroids had scarring limited to the drainage tube inserted site (Fig. 3). The mean mass size for the 11 patients who showed complete response to wide local excision was 3.1 cm, whereas the mean mass size for the nine who required steroid treatment after wide excision was 8 cm.

## Discussion

We performed a retrospective chart review of patients with a definitive diagnosis of GLM treated at a single breast clinic over eight-year period. We analyzed the clinical features, disease manifestations, treatment modality, and treatment outcome in each case. We aimed to evaluate the efficacy of surgical wide excision and drainage with steroid therapy for the treatment of GLM in order to assist treatment planning in this patient population. Though there were some limitations in this study in the fact that it is a retrospective study in a single institution’s data, it is worthy in view of that our population represents inner-city populations of Korea.

GLM presents with symptoms of breast inflammation such as localized warmth and abscess. Antibiotic therapy is the most common treatment employed until a definitive histological diagnosis of GLM is made. After that, the treatment utilized depends on the treatment modality selected by the patient.

Treatments for GLM include local excision, wide excision, abscess drainage, steroid therapy, and observation. There are on-going debates regarding which is the most appropriate treatment.

Lai et al. reported that of eight patients with GLM followed only with observation for 14.5 months, 50% showed spontaneous complete disease resolution and 50% showed no change in disease status [5]. Further, another study reported that when 20 patients were treated solely with antibiotic therapy for 6 weeks, all patients experienced complete remission without recurrence during an average follow-up period of 15 months [6]. Studies have also reported complete remission in patients receiving only conservative treatment after multidirectional abscess drainage in patients whose morbidities did not allow wide excision or steroid therapy [7]. The natural history of GLM may be self-limited, and expectant management with close surveillance may be the optimal treatment modality.

However, because most cases are accompanied by complications such as an abscess or fistula, using expectant management or antibiotics alone is extremely rare. In the present study, no patient was treated with observation or antibiotics alone. However, among the 14 patients who were treated with steroid therapy following incision and drainage, two patients with persistent lesions on their radiographic examination showed improvement in symptoms, and steroid treatment was discontinued at the patient’s request. These patients did not develop new symptoms or complications over 3 years of follow-up period. Thus, expectant management can be a treatment option after treatment for the initial symptoms.

Surgical intervention, including wide local excision, is the oldest and traditional treatment modality. The recurrence rate following local excision is reported to be 23 ~ 50% [2, 4], and consequently, wide local excision that includes the fistula tract has gained acceptance in recent years [8]. Surgical excision showed a fast recovery rate, a high success rate (90.3%) and low recurrence (8.7%) according to Hur et al. [9]. Moreover, in a recent study by Hakan et al. [10], among 77 patients histologically diagnosed with GLM, localized recurrence was found in 9 of 44 patients treated with steroids alone while no recurrence was found in 31 patients treated by surgical interventions such as wide local excision or mastectomy, and there was a difference in patient recovery period (6 months and 1 month, respectively). However, surgical treatments may result in delayed wound healing and excessive scarring left on the breast. In the present study, 11 out of 20 patients who received wide excision recovered completely without any additional drug therapy. These 11 patients had GLM localized to a single quadrant with limited extent and none of these patients experienced a recurrence, but all had excessive scarring and breast deformation. Moreover, following wide excision, 45% (9/20) required additional steroid therapy for delayed wound healing and fistula formation, and after complete recovery, five patients showed recurrence. These were the cases with disease progressing to the subareolar area or other quadrants, and it seemed that even if wide excision apparently accomplished complete removal, there might be residual disease that led to recurrence. Therefore, for extensive GLM that infiltrates beyond the subareolar area, extensive surgical excision should not be attempted and may lead to unnecessary breast deformation, excessive scarring, and costly wound care treatments.

Many studies have concluded that GLM is caused by an autoimmune disease and steroid therapy is a common treatment as a result. The use of corticosteroids for the treatment of idiopathic granulomatous mastitis (IGM) was first proposed by DeHertogh et al. in 1980 [11]. However, the optimal dose and duration of steroid administration has not been established. Previous studies suggested an initial treatment dose of 30–60 mg/day of prednisone which was slowly tapered over several weeks. More recent studies have reported favorable outcomes using short-term, low-dose steroid therapy. In the present study, treatment was initiated with a dose of 20 mg/d. This was tapered over an average of 3.2 months (1 - 6 months) based on the patient’s symptoms. In their retrospective study, Hovanessian-Larsen et al. compared groups of patients with confirmed GLM treated with either steroid or antibiotic alone and reported that 77% (10/13) of the steroid group showed clinical improvement whereas only 5% (2/38) in the antibiotic group showed improvement [3]. In many studies, steroids were used for adjuvant therapy following surgical excision or drainage [12, 13], and the results of using steroids alone have not been widely reported. In a recent study by Sakura et al. [14], eight patients with a breast mass diagnosed as GLM were treated with steroids alone or with concomitant antibiotics if signs of infection were present. All but one patient, who underwent excision for pain, showed complete improvement after steroid therapy for 4 -10 months. Other reports have indicated favorable outcomes following co-administration of oral steroid together with topical steroids for cutaneous diseases [15]. Steroid alone may be the main treatment modality in cases without abscess or infection.

There were some studies comparing surgery and steroid therapy for treating GLM, summarized in Table 2. Oran et al. [16] reported that steroid therapy group and surgery group showed comparable treatment outcome in view of recurrence (20% versus 16.7%, respectively). Rather, Akahane K et al. [17] reported no recurrence in steroid group, whereas100% (2/2) in surgery group showed recurrence, suggested steroid therapy as first choice of treatment in GLM.

**Table 2:** Summary of available literature comparing surgery and steroid therapy for treating GLM

Authors	Number of patients	Median F/U (month)	Treatment Modality (n)		Duration of steroid treatment (mo)	Recurrence (n, %)	Management of recurrence	Second Recurrence
Kok et al. 2010, [2]	43	15	Surgery^a^	(40)		10 (23%)	Excision (9) Excision + steroid (1)	2
			Steroid	(3)	1	-		
Yabanoglu H et al. 2015, [10]	77	13.5	Surgery	(33)	1	0		
			Steroid	(44)	6	9 (20.4%)		
Oran et al. 2013, [16]	46	35	Excision	(18)		3 (16.7%)	Steroid (1), Excision + steroid (2)	
			Steroid	(25)	4	5 (20%)	Steroid (2), Steroid + excision(2), Excision (1)	
			Steroid + Excision	(3)		-		
Akahane K et al. 2013, [17]	12	22	Surgery	(2)		2 (100%)		
			Steroid	(10)	5	-		

^a^Includes excision (18), incision and drainage (21), wide local excision (1)

In the present study, there was no group given steroid alone as the initial treatment. But 14 patients who were treated with steroids following incision and drainage showed favorable treatment outcomes, resulting only one recurrence. Although eight out of 14 patients had localized disease limited in single quadrant, they showed favorable outcome without surgical excision and left favorable cosmetic outcome. Moreover, six patients who developed recurrence also treated with steroids alone and experienced complete response. But, long-term steroid use can lead to glucose intolerance and Cushinoid features such as weight gain, depression, cataracts, and increased susceptibility to infections [13]. Some have reported combining methotrexate and steroids for the treatment of GLM, but the long-term follow-up results have not yet been reported [18–20]. In these reports, immunosuppressive therapy was found to be effective in reducing steroid use, controlling the inflammatory process, and preventing further complications.

Our retrospective analysis included patients with GLM during 8 years, who were followed up for median 45.5 months. Based on the literature and personal experiences, recurrences may present several months to years after initial treatment. We excluded patients with GLM diagnosed in the recent 3 years to identify the long-term outcome. Furthermore, the types of treatment widely vary. Complete wide excision was the preferred therapeutic modality in earlier years and steroid therapy or nonsurgical conservative treatment is the preferred therapeutic modality in recent years. This difference is due to literary reports and treatment behaviours in those respective periods. Though the treatment periods were different in two group, clinical presentation, and follow up periods were similar. Thus, we could compare the therapeutic outcome of wide excision and steroid with drainage.

In conclusion, the proper treatment method must be chosen for each individual patient. In cases of small localized disease that has not infiltrated the subareolar area, wide excision and steroid with drainage can be chosen by patients’ preference. Doctors should provide information about the pros and cons of each treatment modalities including cosmetic outcome. But, in cases of diffuse disease involving subareolar area and multiple quadrants, better outcomes can be achieved using incision and drainage followed by steroid therapy for patients with an abscess and steroid therapy alone for patients without an abscess. Further, incision and drainage is also invasive procedure that can make scarring and need anesthesia, repeated ultrasound guided aspirations for collections and steroid use can be enough for patients with an abscess. More studies comparing incision and drainage and ultrasound guided aspiration for treating GLM with abscess is needed.

## Conclusions

Our retrospective analysis demonstrated that steroid therapy with or without abscess drainage may be the first choice in most cases of GLM. Surgical wide excision can be selected only in limited disease and in patients with contraindication to steroid therapy.

## Abbreviations

GLM: Granulomatous lobular mastitis
TB: Tuberculosis
